# Future Meetings of the U. S. V. M. A.

**Published:** 1897-01

**Authors:** 


### FUTURE MEETINGS OF THE U. S. V. M. A.

THE division into sections for a part of the time fixed for our annual convention would give opportunity for special work of much value. A section comprised of those interested in agricultural college work would naturally draw together those associated with the organization of our veterinary faculties and result in a better understanding of the work to be accomplished, all of which could be best promoted in the way suggested. The laws recently enacted in New York State must necessarily prepare their students for better scientific work, and commercial institutions in scientific fields must go to the wall. So our national organization can well favor every one of these new avenues that tend to broaden and make better the science we represent, and, while aiding and supporting every institution whose aim is higher education, we still remain in that neutral field best fitted for us, of not dictating what schools shall do. Let the officers of the U. S. V. M. A. take up this suggested change.

